# Human immunodeficiency virus and antiretroviral therapy-mediated immune cell metabolic dysregulation in children born to HIV-infected women: potential clinical implications

**DOI:** 10.3389/fimmu.2023.1182217

**Published:** 2023-06-07

**Authors:** Hope Mataramvura, Madeleine J. Bunders, Kerina Duri

**Affiliations:** ^1^ Immunology Unit, University of Zimbabwe Faculty of Medicine and Health Sciences (UZ-FMHS), Harare, Zimbabwe; ^2^ III. Medical Department, University Medical Centre Hamburg-Eppendorf, Hamburg, Germany; ^3^ Department of Virus Immunology, Leibniz Institute of Virology, Hamburg, Germany

**Keywords:** early life antiretroviral therapy exposure, mother to child transmission of HIV, immune-metabolic dysregulation, mitochondrial toxicity, immunity

## Abstract

Commencing lifelong antiretroviral therapy (ART) immediately following HIV diagnosis (Option B+) has dramatically improved the health of HIV-infected women and their children, with the majority being of HIV-exposed children born uninfected (HEU). This success has led to an increasing population of HIV-infected women receiving ART during pregnancy and children exposed to ART *in utero*. Nonetheless, a small proportion of children are still infected with HIV (HEI) each year. HEI children suffer from reduced immunocompetence and host-defence, due to CD4+ T lymphocyte depletion, but also dysregulation of other immune cells including CD8+ T lymphocytes, natural killer (NK) cells, macrophages including B lymphocytes. Furthermore, although HEU children are uninfected, altered immune responses are observed and associated with increased vulnerability to infections. The mechanisms underlying immune dysregulation in HEU children remain poorly described. Building on early studies, emerging data suggests that HIV/ART exposure early in life affects cell metabolic function of HEU children. Prenatal HIV/ART exposure has been associated with dysregulation of mitochondria, including impaired DNA polymerase activity. Furthermore, dysregulation of oxidative phosphorylation (OXPHOS) causes a decreased generation of adenosine triphosphate (ATP) and increased production of reactive oxygen species (ROS), resulting in oxidative stress. These altered metabolic processes can affect immune cell viability and immune responses. Recent studies have indicated that immune-metabolic dysregulation may contribute to HIV-associated pathogenesis and clinical observations associated with HIV and ART exposure in HEU/HEI children. Given the critical role metabolic processes in immune cell functioning, immune-metabolic dysregulation in HEU and HEI children may have implications in effective host-defence responses against pathogens, as well as efficacy of standard ART regimens and future novel HIV cure approaches in HEI children. At the same time, targeting metabolic pathways of immune cells may provide safer and novel approaches for HIV cure strategies. Here, we review the current literature investigating immune-metabolic dysregulation in paediatric HIV pathogenesis.

## Introduction

In 2020, 37.7 million people were living with HIV/AIDS (PLWHA), and of these, 1.7 million were children aged from 0 to 14 years. During the same period about 150,000 new infections occurred in children aged 0 to 9 years with up to 46% of the HIV-exposed infected (HEI) children lacking access to antiretroviral therapy (ART) (1). Without ART 50% of the HEI children are expected to succumbed to HIV by the age of 2 years as a result of adverse birth outcomes like prematurity, low birth weight (LBW) and HIV-associated impaired immunity to bacterial and viral infections early in life (2). Over the past decade the successful roll-out of ART for the prevention of mother-to-child transmission (PMTCT) of HIV has significantly reduced vertical transmission (3).

More than 90% of pregnant women globally now have access to PMTCT of HIV interventions (3). As a result, the population of maternally HIV-exposed uninfected (HEU) children is increasing with approximately a global population of 14.8 million HEU children in 2018 (4) and this figure continues to increase. However, some of the children born to HIV-infected women are still vertically infected annually with 160000 new infections recorded in the 0 to 14 year age group in 2020 (5). Dual nucleos(t)ide reverse transcriptase inhibitors (NRTIs) backbone in combination with a non-NRTI (NNRTI) or an integrase strand transfer inhibitor (INSTI) are the currently recommended ART regimens to prevent vertical transmission (6). ART can cross the placenta and detectable in cord blood (7). NRTI’s are known to dysregulate the proper functioning of mitochondria (8, 9), and concerns have been raised regarding the long term impact of *in utero* ART exposure and in early life after birth.

Despite having significantly improved the quality of life of PLWHA, ART has also been associated with a range of adverse effects both in children and adults, particularly metabolic changes have been reported (9–11). HEU children are exposed to maternal HIV and ART *in utero*, continuing throughout the breastfeeding period. Both HEU and HEI children present with higher morbidity and mortality rates as a result of altered immune responses when compared to their HIV unexposed uninfected (HUU) peers born to HIV uninfected women (12–14). Furthermore, reduced growth is observed in the early years of life in HEU and HEI children compared to their HUU counterparts (15).

Cell metabolism is critical for the functioning of cells, organs and overall human development. Metabolic dysregulation can affect the transport, utilization or storage of metabolites such as glucose, lipids and amino acid within cells. HIV infection independent of ART can induce changes in glucose, lipid and amino acid metabolism. Even in the presence of ART these observations are not fully corrected (16). Metabolic dysregulation may have clinical implications, including host-defense against pathogens, tissue growth and development, and neurological functioning. Furthermore, as proper metabolic functioning is critical for effective immune responses, hence metabolic dysregulation may have implications for the development of potential future HIV cure approaches for HEI children.

The current approach to improve health of HEI children relies on early initiation of ART (17, 18). This strategy aims to prevent the establishment of large viral reservoirs and maintenance of host immunocompetence. HIV cure approaches target the virus but also rely on a good immune system, therefore an effective host immune responses would be critical for the success of these approaches. Metabolic impaired immune cells may render potential HIV cure strategies less effective (19, 20). At present our knowledge of metabolic dysregulation and consequences of impaired immune function in HEI children is limited. However, the topic has been investigated in more depth in adults. These studies in adults may inform paediatric studies in the development of potential strategies to restore metabolic homeostasis and improve cure approaches. A better understanding of metabolic dysregulation in HEI children may also help to identify pathways that provide targets to ameliorate long-term consequences of growth impairment and tissue health. In light of this, it is crucial that paediatric studies investigating novel HIV cure strategies take into account metabolic functioning of immune cells, including the mitochondria.

## Mitochondria are regulatory hubs for cell metabolism and functioning

Mitochondria are organelles in the cell, which serve a variety of critical functions in eukaryotic cells. The primary function of the mitochondria is energy production in the form of adenosine triphosphate (ATP) mainly through oxidative phosphorylation (OXPHOS)/electron transport chain (ETC) and the citric acid cycle. Mitochondria are furthermore involved in regulating cellular metabolism, cell signalling and maintenance of cell redox state (21, 22). Disruption of mitochondrial functioning can result in lack of energy and increased reactive oxygen species (ROS), with both triggering processes ultimately resulting in cell senescence and death (23). In the context of immune responses during activation, immune cells shift from a resting to an active state. This process requires energy in the form of ATP (24), underscoring the importance of ATP in activating immune responses. Furthermore, ROS are potent activators of proinflammatory signalling pathways. Thus the cell’s metabolism hubs, the mitochondria, is therefore central in the establishment and maintenance of immune responses (25, 26), processes that are compromised in HIV infection.

### HIV-associated alterations in mitochondrial functioning

In HIV infection, both the virus and ART alter mitochondrial functioning which can have consequences for the health of PLWHA (27). HIV can directly induce reprogramming of mitochondrial functioning through its proteins including *Tat, gp120*, *Nef* and *Vpr. Tat* and *Vpr* thereby affecting the mitochondrial transmembrane potential, resulting in mitochondrial swelling and cell apoptosis (28, 29). Gp 120 furthermore affects mitochondrial fusion and mitochondrial size (30), as well as the upregulation of glycolysis as indicated by a high extracellular acidification rate (31). Furthermore, HIV proteins can also induce production of ROS through Env-mediated autophagy of peroxisomes (32) and Tat-induced DNA damage (29), in the process dysregulating oxidative stress-regulating pathways (33). These processes regulate immune responses by activating inflammatory responses through the Nod-like receptor (NLR) family of pyrin domain containing 3 (NLRP3) inflammasome that mediate antiviral responses (34, 35). Continuous signalling, however, in the context of chronic HIV infection can therefore result in prolonged signalling and inflammation associated HIV immunopathogenesis.

### ART-associated alterations in mitochondrial functioning

Not long after the introduction of NRTIs their mitochondrial toxicity became apparent (9, 36, 37). Zidovudine, a NRTI, has long been shown to cause metabolic dysregulations. A study by Blanche et al. demonstrated a low mitochondrial complex I (CI) and complex IV (CIV) activity in children exposed to AZT *in utero* and after birth (9) (**Table 1**). Since then, NRTIs, NNRTIs and protease inhibitors (PIs) have all been reported to induce mitochondrial dysfunction with more pronounced effects observed of NRTIs. NRTIs were initially thought to affect mitochondrial functioning by acting as substrates to polymerase gamma, thereby disrupting mtDNA synthesis. However, recent studies indicate alternative effects that also alter mitochondrial functioning (8, 57).

**Table 1:** Metabolic processes in HEU and HEI cohorts compared to HUU controls.

Metabolite/organelle	HEU children	HEI children	Ref
**Glucose**	-HOMA-IR comparable to HUU - paucity of data on fasting blood glucose	-HOMA-IR > 2.5 indicating insulin resistance -impaired fasting blood glucose (>100mg/dL)	(38–43)
**Amino-acid**	-Increased acyl-carnitine profile	-Reduced serum carnitine levels	(44–48)
	- correlation of branched amino acids with insulin resistance	- paucity of data	
	-increased methionine-sulfone significant in HEU with long-term ART exposure	-paucity of data	
**Lipid**	-Increased plasma concentration of triglycerides	-Increased total cholesterol, low-density lipoproteins and triglycerides	(44, 45, 49, 50)
	-Increased saturated lysophospholipids and decreased unsaturated lysophospholipids	- paucity of data	
	-increased sphingolipids and ceramide	-paucity of data	
**Mitochondria**	-Mitochondrial morphological damage and reduced mtDNA levels	-Reduced mtDNA levels	(9, 51–54)
	- Reduced OXPHOS protein levels and enzyme activity	- Reduced OXPHOS complex I and IV protein levels and activity	(51, 55, 56)

HOMA-IR, homeostatic model assessment for insulin resistance; mtDNA, mitochondrial deoxyribonucleic acid; OXPHOS, oxidative phosphorylation; HUU, HIV-unexposed uninfected; HEU, HIV-exposed uninfected; HEI, HIV-exposed infected.

Furthermore, NRTIs can act as DNA chain terminators through their incorporation into the mtDNA leading to an aborted replication with a reduced mtDNA copy number per cell (58). Although initial studies have focused on Stavudine and Zidovudine a study by Zhao and colleagues showed that the transcription factor SSBP1 and the mitochondrial DNA helicase were both down-regulated by Tenofovir which may reduce mtDNA (8). Tenofovir is still widely used (6). PIs directly inhibit cell metabolism through decreasing the mitochondrial membrane potential which is pivotal in ATP production, but this effect may vary with cell type (59, 60). Efavirenz, like HIV infection, inhibits CI in the human ETC whilst murine studies have shown that Efavirenz inhibits CIV resulting in decreased ATP production and increased ROS production (61). It has been hypothesized that OXPHOS dysfunction is the mechanism behind lactic acidosis observed among HEI children on ART (62). A recent study reported elevated levels of methionine-sulfone, a result of increased methionine oxidation by ROS, in HEU born to women who initiated ART preconception when compared to those who initiated ART post-conception (44). This is indicative of long-term ART exposure being associated with oxidative stress in HEU.

Several studies have shown a decrease in mitochondrial CI and CIV protein levels in peripheral blood mononuclear cells (PBMCs) of PLWHA on ART, correlating with disease severity as assessed by the CD4/CD8 ratio (62–64). One of these studies showed that the levels of PBMCs CI and CIV protein levels were inversely related to plasma inflammatory markers (monocyte chemotactic protein-1, myeloperoxidase, serum amyloid A, serum amyloid P, soluble adhesion molecules) and inflammatory intermediate monocyte frequencies (64, 65). In return inflammatory molecules can further enhance mitochondrial dysfunction, increasing ROS production thereby driving enhanced dysregulation and inflammation. In sum, HIV and ART can alter mitochondrial functioning through different pathways of dysregulation of the cell metabolism and downstream immune responses.

### Glucose metabolism

Glucose is the primary source of energy in mammalian cells and is crucial in cell metabolism as it is a biosynthetic precursor which feeds into glycolysis and indirectly into the pentose phosphate pathway, Krebs cycle and OXPHOS (66, 67). These pathways generate ATP, which transfers energy by releasing a phosphate group (68). In immune cells, processes such as cell movements and effector functions all require energy in the form of ATP (69). A variety of infections such as HIV can alter transporters and enzymes involved in glucose metabolism which are known to affect the functioning of immune cells (19, 20).

#### HIV and glucose metabolism

HIV has been reported to interfere with energy synthesis pathways, causing an increased expression of glucose transporters. Glucose transporter-1 (GLUT-1), glucose transporter-3 (GLUT-3) which facilitates the transport of glucose across the plasma membrane, including glucose transporter-4 (GLUT-4), glucose transporter-6 (GLUT-6) and hexokinase 1 (HK1) are up-regulated in adult HIV-infected CD4+ T lymphocytes (19, 20). A study by Mason and colleagues demonstrated that GLUT-1 expression positively correlated with mitochondrial mass and mitochondrial membrane potential (70). Another study in adult PLWHA demonstrated that HIV infection caused an increase in the uptake of a fluorescent glucose analogue, 2NBDG, by infected monocytes (71). Remarkably, upregulated glucose metabolism has been associated with CD4+ T lymphocytes apoptosis although it is unknown whether these changes may be involved in the decrease of CD4+ lymphocytes in HIV infection (72, 73).

In addition, the changes in glucose transport have been associated with increased levels tumour necrosis factor (TNF) production in HIV-infected CD4+ T lymphocytes (73). Long-term viral replication and dysregulation of glucose levels have been associated with inflammation which possibly contribute to chronic immune activation in adult HIV patients. Further studies are warranted to investigate the regulation of these glucose transporters in HEU and HEI children. However, such studies in the context of obesity have shown that long term increases of glucose levels are associated with chronic inflammation and reduced antigen specific adaptive responses (74, 75).

#### ART exposure and glucose metabolism

Building on the research investigating mitochondrial toxicity of ART, Tenofovir-mediated down-regulation of the mitochondrial chaperone TRAP1 may be involved in the reprogramming of glucose metabolism with increased glycolysis and glycogen synthesis (8). ART has also been shown to induce insulin resistance in PLWHA providing the underlying conditions for the development of type 2 diabetes, which is common among this population (76). Insulin resistance, measured using the Homeostatic Model Assessment of Insulin Resistance (HOMA-IR), is also common among HIV-infected children on ART. A recent study observed insulin resistance in 20% of the HEI children with clinical end stage of the disease and long-time on ART (38, 77) (**Table 1**). Changes in glucose transport and metabolism contribute to impaired insulin secretion by beta cells and altered glycogen synthesis in hepatocytes which significantly increase the risk of developing type 2 diabetes among PLWHA on ART (78). There is urgent need for comprehensive research on the glucose metabolic dysregulation in HEU children with *in utero* and breast milk exposure to maternal HIV/ART versus HEI children with additional direct exposure to HIV and ART as these two groups may have distinct characteristics which inform on their specific health needs.

### Lipid metabolism

Next to glucose, lipid metabolism has been shown to be critically altered in PLWHA (79, 80). Lipids are organic compounds which play vital roles in the human body. These include storage of energy, as structural components in plasma membranes, as biomarkers, as hormones and in cell signalling in mammalian cells (81). Lipids can also be used as an alternative source of energy by the cells. The human body utilizes different types of lipids. Triglycerides are used to store energy in lipids and are increased in obese individuals (82). Studies focusing on obesity have shown that increased levels of triglycerides alter immune responses associated low level immune activation but reduced specific immune responses to pathogens or vaccines (74, 75).

### HIV and lipid metabolism

HIV infection, in the absence of ART, has been shown to induce alterations in serum lipid profiles (83). HIV-associated dyslipidemia is characterized by high total cholesterol, triglyceride levels and low high-density lipoprotein cholesterol. HIV infection furthermore affects reverse cholesterol transport causing a decrease in cholesterol levels and an increase in triglycerides (84). Furthermore, cytokines can have a direct effect on lipid metabolism altering lipid profiles through modifications of lipid processing and transport in chronic inflammatory states (45, 78). HIV infection in macrophages causes an increase in lipid uptake as indicated by fluorescent lipid dye; BODIPY intensity, resulting in lipid accumulation and increased mitochondrial size, but, decreased functioning (85). Abnormal lipoprotein profiles and inflammatory markers have been shown in HIV-infected children and adolescents (86–88). These metabolic changes and chronic inflammation may have clinical implications for cardiovascular diseases (CVD) as they mature into adulthood.

The dysregulation of mitochondria also has repercussions for lipid metabolism. The excess ROS produced from mitochondrial dysfunction react with polyunsaturated fatty acids during lipid peroxidation, and levels are increased in plasma of children born to HIV-infected women compared to those born to healthy women (45). The products of lipid peroxidation, malondialdehyde (MDA) and 4-hydroxynonenal, furthermore can alter the integrity of cell membranes (89). Mitochondrial membrane lipid peroxidation destabilizes the membrane structure and changes integrity, resulting in the mitochondrial permeability transition pore formation, loss of the mitochondrial transmembrane potential, release of cytochrome *c* and eventually cell death (63, 90). Thus, mitochondrial dysregulation via altered lipids may further aggravate mitochondrial dysfunction, with consequences for energy production and inflammation.

#### ART exposure and lipid metabolism

PIs have long been associated with dyslipidemia in PLWHA (91). Unresolved dyslipidemia increases the risk of developing CVDs (92, 93). Elevated levels of triglycerides are observed with PLWHA on PIs (93). Exposure to ART particularly Ritonavir-boosted Lopinavir has been associated with hypertriglyceridemia, previously demonstrated in adults (94), and decreased phospholipids in children (45). Furthermore, Efavirenz, has been shown to activate AMP-activated protein kinase, subsequently promoting lipid accumulation in the cytoplasm associated with the increase in mitochondrial mass (95). *In utero* ART-exposed HEU-children have an altered lipid profile compared to HUU children (45). They have increased plasma concentrations of triglycerides, saturated lysophospholipids and decreased levels of unsaturated lysophospholipids compared to HUU children (**Table 1**). Another study reported an altered sphingolipid-ceramide ratio in HEU with long-term ART exposure than in those with medium, short or no exposure (44). This is indicative of an altered lipid metabolism with increase in the duration of *in utero* ART exposure. Phospholipids are important components of the cell membranes and have anti-inflammatory properties (96). The increased levels of triglycerides and subsequent decreased levels of phospholipids are important triggers for the unfolded protein response (UPR) of the endoplasmic reticulum and the pro-inflammatory milieu in early life ART-exposed HEU-children (45).

### Amino acid metabolism

Amino acids are organic molecules, which act as building blocks for protein synthesis, e.g. enzymes. Metabolism of proteins to generate energy only occurs in situations of low carbohydrate or lipid intake such as during starvation or in situations of high energy demand (97, 98). Furthermore, glutamate is critical for the functioning of activated T lymphocytes as it provides a fuel source which sustains mitochondrial OXPHOS (99). In NK cells amino acids play less of a role as energy substrates. However, amino acids do regulate NK cell functioning. Glutamine-regulated expression of the transcription factor cMyc is critical in controlling NK cell proliferation and effector functions (100, 101).

#### HIV infection and amino acid metabolism

Recent studies have reported an association between CD4+ T lymphocyte counts in PLWHA and serum glutamine indicating that this amino acid is utilized by cells as an energy source during HIV infection (72, 102). In addition, macrophages during HIV infection utilize glutamine as an alternative source of energy (19). In one study of HIV-infected macrophages showed plasticity in fuel usage by switching the source of energy when either glucose, fatty acid or glutamine was inhibited (19). Inhibition of ASCT2, a glutamine transporter, resulted in a decrease in the number of surviving HIV-infected macrophages indicating glutamine as the primary energy source in infected macrophages, hence absence of glutamine may restrict HIV infection (19).

Other amino acids are involved in the functioning of mitochondria. Cysteine and methionine residues on amino acids are easily oxidized by ROS to form disulphides (103, 104). Mitogen activated protein kinase (MAPK), a signalling complex which regulates proliferation and aerobic glycolysis, is rich in cysteine residues and formation of disulphides in the protein structure results in a dysregulated cell signalling function (104). The metabolic functioning of the mitochondria is reflected by the levels of intermediary metabolites such as acyl carnitines which are formed during fatty acid oxidation. Studies in ART-exposed HEU-children demonstrated an altered plasma metabolite profile especially acyl-carnitine profile and branched amino acids compared to HUU children (45, 46) (**Table 1**). One of the studies showed that postnatal exposure to Zidovudine had a greater impact on the metabolic profiles than Nevirapine (46). HIV/ART exposure has also been associated with an abnormal phenylalanine metabolism and increased phenylalanine in the circulation (57). Phenylalanine can cross the placenta and even small increases in plasma phenylalanine levels have been reported to be associated with decreased head circumference and birth length (105). Additionally, HIV-mediated changes in tryptophan metabolism have been shown to result in suppressed serotonin production and increased kynurenine production through the upregulation of indole amine 2,3-dioxygenase (IDO) expression that degrades the tryptophan (57). A study by Babu and colleagues demonstrated that an increase in kynurenine/tryptophan ratio and lower levels of serotonin in more than half of their cohort of PLWHA and both were associated with neurocognitive impairment and HIV-associated neurological disorders (106). Serotonin is essential in neurons activation and altered levels may result in neurocognitive impairment (107). Such alterations in pregnancy may affect the neurological development in the foetus.

**Figure 1:**
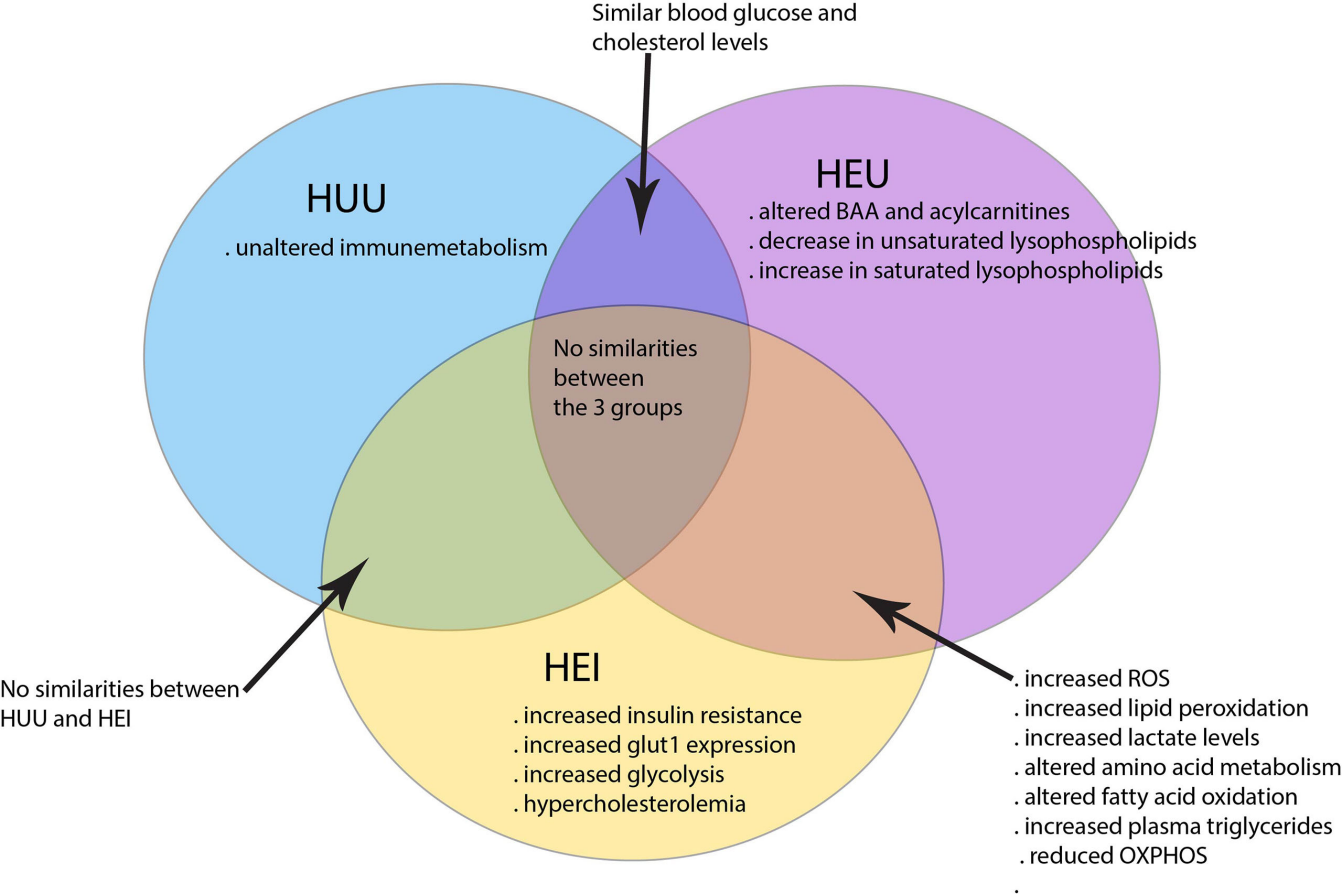
Immunometabolism distinguishing HEI, HEU and HUU children. HEI and HEU children share many similarities in altered cell metabolism as compared to the HUU children.

Taken together, HIV and ART can in many ways impact the cell metabolism with consequences for immunity against pathogens, chronic inflammation and dysregulation of energy metabolism to sustain development during infancy. The HUU children have clearly distinct immunometabolism than in HEI and HEU children as illustrated in **Figure 1**.

Several studies have linked the metabolic dysregulations in HEI children to clinical implications such as a higher risk of CVD in HEI when compared to uninfected peers (108–110). Altered sphingolipid-ceramide profiles as reported in HEU children with long-term ART exposure (44) may predispose them to age-related neurological disease (111, 112). The latter is a CVD biomarker hence like HEI, HEU with long-term ART exposure may be at a higher risk of CVD compared to HUU children (113). However, most studies in HEI and HEU cohorts describe an increased mortality and morbidity and/or immune cell phenotypes but not linking them to metabolic assays (114, 115). This has left any association between dysregulated metabolism and clinical implication poorly described creating a need for such studies (116, 117).

Below we describe the current understanding of altered immunity in HEI and HEU children and how metabolic dysregulation can contribute to these observations.

### Altered immunity in children born to HIV-infected women

#### Infant vaccine responses

At birth children initially rely on their innate immune responses and passive acquired immunity from the mother thanks to the transfer of maternal antibodies from the mother to the foetus during pregnancy. During childhood immune maturation in response natural infections and vaccinations results in the acquisition of effective adaptive responses. In HEU-children antibody mediated passive as well as vaccine induced immunity is affected with HEU children showing altered vaccine-specific responses compared to HUU as previously reviewed (118). Although data has been conflicting overall HEU and HEI have lower antibody responses to vaccines shown by the low titres of anti-tetanus, measles, diphtheria, pertussis and hepatitis B surface antigen antibodies as compared to HUU (118–120), whereas conflicting reports have been published for BCG, pneumococcus and pertussis vaccines (121, 122). In HEI children the low CD4+ lymphocyte counts, further critically impairing the induction and maintenance of immunological memory to vaccines and infections. This indicates HIV-mediated poor quality and quantity of humoral responses among this group (118).

### Innate immunity in children

Innate immunity by NK cells and macrophages plays a crucial role in responses to infections early in life before adaptive immune responses have matured. Exposure to viruses such as HIV is associated with NK cell dysfunction (123). HIV infection in children is associated with lower NK cell numbers and altered phenotype and effector functions of NK cells in HEI children (124, 125). Exposure to HIV is furthermore associated with reduced cytolytic potential of NK cells in HEI and HEU children as indicated by the lower expression of perforin compared to HUU children (124, 125).

The expression of CD69, which is upregulated upon cell activation, is increased on NK cells in HEI compared to HUU children. This finding suggest that NK cells undergo increased activation in HEI compared to HUU children in the early months of life (125). At the same time, NK cells of HEI children express higher levels of NKG2A and KIRs inhibitory markers (126), which may inhibit NK cell activation against virus- infected cells such as HIV-infected cells. Taken together, NK cells have an altered phenotype associated with reduced cytotoxic characteristics in HEU and HEI-children, however the question remains whether metabolic changes in NK cells may underlie these observations (127).

Macrophages and dendritic cells (DC) are innate immune cells that act as antigen presenting cells (APC) and shape the adaptive immune system during an infection. Macrophages express chemokines receptors, CCR5 and CXCR4 that are co-receptors in HIV infection. HIV infection has been reported to induce changes in macrophage polarization state depending on the stage of the infection (128). Due to their plasticity during HIV infection, macrophages can polarize towards different phenotypes with increased or decreased antiviral capacity (128, 129). With regards to DCs HEU-children at birth have a significantly higher percentages of myeloid dendritic cells (mDCs) than HUU-children (130), however, the frequencies of both mDCs and plasmacytoid dendritic cells (pDCs) remain similar throughout the first year of life (130, 131). Upon stimulation with lipopolysaccharide (LPS) mDCs from HEU-children showed increased upregulation of CD80, CD86 and programmed cell death ligand 1 (PD-L1) while the mDCs from HUU children mostly upregulated PD-L1. Furthermore, mDCs of HEU-children at birth showed a higher responsiveness to LPS and polysaccharides from *Atractylodes macrocephala* (PAM) stimulation by secreting TNF, IL-6 and IL-12 compared to HUU children (130). Beyond 6 weeks of life mDCs responses to bacterial ligands became comparable among the two groups. In sum, innate immune cells are affected in HEU-children especially shortly after birth.

Several studies have shown that HIV-induces a switch of source of energy in innate immune cells such as macrophages (85, 132). Glucose and fatty acid utilization are reduced in adult HIV-infected macrophages while glutamine uptake is increased (19, 133). There is paucity of data regarding the implications of HIV/ART-mediated metabolic dysregulation in macrophages and DCs on the immune responses in children. Some adults’ studies have shown that HIV infection mediates IFN responses and toll-like receptor (TLR) engagement resulting in intracellular IDO expression in macrophages and DCs. This increases the levels of circulating immunomodulatory tryptophan catabolites like kynurenine and quinolinic acid which have been associated with higher production of inflammatory cytokines and increased immune activation (134). In addition, the higher glycolytic flux in infected macrophages leads to increased production of pro-inflammatory cytokines such as TNF and IL-6 further fueling chronic inflammation in PLWHA (132). These studies were in adults and the impact of such metabolic dysregulations in children are yet to be described.

Furthermore, HIV induces mitochondrial enlargement in infected macrophages. A possible cause of mitochondrial enlargement in HIV infected cells is lipid accumulation in the mitochondrial matrix (85). The clinical implications of this is not clear. In addition to enlargement, mitochondria of infected macrophages have significantly lower basal oxygen consumption rate and response to oligomycin, an ATP synthase inhibitor (85, 135). This indicates HIV-mediated mitochondrial dysfunction in infected macrophages that may persist in HEI children whereas the effects of maternal HIV may only be affecting macrophages and DCs in HEU children for shorter periods.

### Adaptive immunity in children

T lymphocytes serve as effector cells of adaptive immune responses. CD4+ T lymphocytes are susceptible to HIV infection due to their expression of CD4 and CXCR4 and CCR5 (co)-receptors. Next to the challenge of HIV associated cell death of CD4+ T lymphocytes in HEI children, chronic immune activation further affects cell populations in children and in adults (73, 136). Metabolic changes in T lymphocytes have been described in PLWHA, e.g. the glucose transporter GLUT1 is upregulated. An inverse association between the number of CD4+GLUT1+ T lymphocytes and CD4+ T lymphocyte counts has been reported (68, 73) indicating an association between cell death and glucose metabolism in CD4+ T lymphocytes.

In children, HIV infection is associated with an approximately 1.5 times higher frequencies of programmed cell death 1 (PD1)+ memory CD4 T lymphocytes compared to uninfected children (137, 138). This dysregulation was shown to be partially reversed by ART as the frequencies decreased upon ART initiation however, remaining higher than in uninfected children (138). High frequencies of programmed cell death protein 1 (PD1)+ memory CD4+ T lymphocytes predicts lower effector capacity. CD8+ T lymphocytes are important in the control of HIV infection (139–141). However, prolonged activation of CD8+ T lymphocytes results in cell exhaustion. Inhibition of PD1 on CD8+ T lymphocytes reverses cell exhaustion and restores anti-viral capacity *in vitro*, at one point PD1 inhibition has been suggested as a potential target in HIV cure, however adverse effects are not negligible therefore making this currently a limited option for cure (142).

Activation of CD4+ T lymphocytes in response to antigen recognition presented by macrophages or DCs during HIV infection enhances glycolysis which in turn facilitates HIV replication and establishment of a large reservoir (72). Unlike in CD4+T lymphocytes, increased glycolytic activity during activation and differentiation in CD8+ T lymphocytes is associated with viral suppression (141). GLUT1 expression is correlated with mitochondrial density and mitochondrial membrane potential (MMP) in CD4+T lymphocytes. Furthermore, the MMP has been positively correlated with ROS production in CD4+ and CD8+ T lymphocytes (70). If let unresolved, ROS can damage the cells reacting to cellular metabolites or DNA as discussed earlier (70).

*In utero* HIV/ART exposure also has an impact on CD4+ T lymphocytes and CD8+ T lymphocytes in HEU-children (143). HEU-children have decreased CD4+ T lymphocyte counts but increased CD8+ T lymphocyte counts (115, 144, 145) and in one longitudinal study has observed altered CD8+ count that persisted until 8 years of age (144). Furthermore, several studies have reported consistent findings of increased activated CD8+ T lymphocyte and memory CD4+CD45RO+ T lymphocytes among HEU-children compared to HUU-children (143, 146). Although CD4+ T lymphocytes in HEU-children have an activated phenotype (CD4+ HLA- DR+ CD38+), IL-2 production was reduced compared to HUU-children (146). The T lymphocytes phenotype in HEU-children reflects to a certain extend the increased activation. Studies on the metabolic dysregulations in T lymphocytes of HEU-children are warranted to understand whether metabolic dysregulation may further underlie these observations.

### Impact of metabolic dysregulation on current HIV cure strategies

As PMTCT strategies are not reaching all women in time, paediatric HIV infection still occurs. Therefore, there is a need for HIV cure approaches in children. The success of an HIV cure depends on the eradication of viral reservoir in latent infected cells. Early ART initiation was one of the first strategies to be tested for a functional cure among children (147–149). The early ART initiation approach aims to limit the size of the viral reservoir.

In efforts to cure HIV, the “shock and kill” approach has been suggested where latent infected cells are reactivated and next are recognised and eliminated by immune cells. Another HIV cure approach is the use of therapeutic vaccination which aims to enhance HIV-specific T lymphocyte responses (149–151). Finally, antibody based approaches have been suggested (152, 153). All the above cure approaches depend on a functional immune system to finally eliminate infected cells. The “surge and purge” approach which combines early ART, passive antibody administration and immune stimulation has been hypothesized to affect reservoir establishment (154). This approach and the “block and lock” may be successful in children with small viral reservoirs by blocking HIV production. In children with large HIV reservoirs it has been suggested that a combination of reversal agent, to reactivate viral expression, and clearance (kick and kill) of the infected cells by the immune system may be successful (155, 156). Considering the metabolic dysregulation of immune cells in HIV infection and upon ART exposure, the immune system of HEI-children is moderately to severely impaired. This then acts as a barrier to the eradication of the virus by the different HIV cure approaches (149).

### Targeting metabolic pathways to improve immune functioning

**Figure 2:**
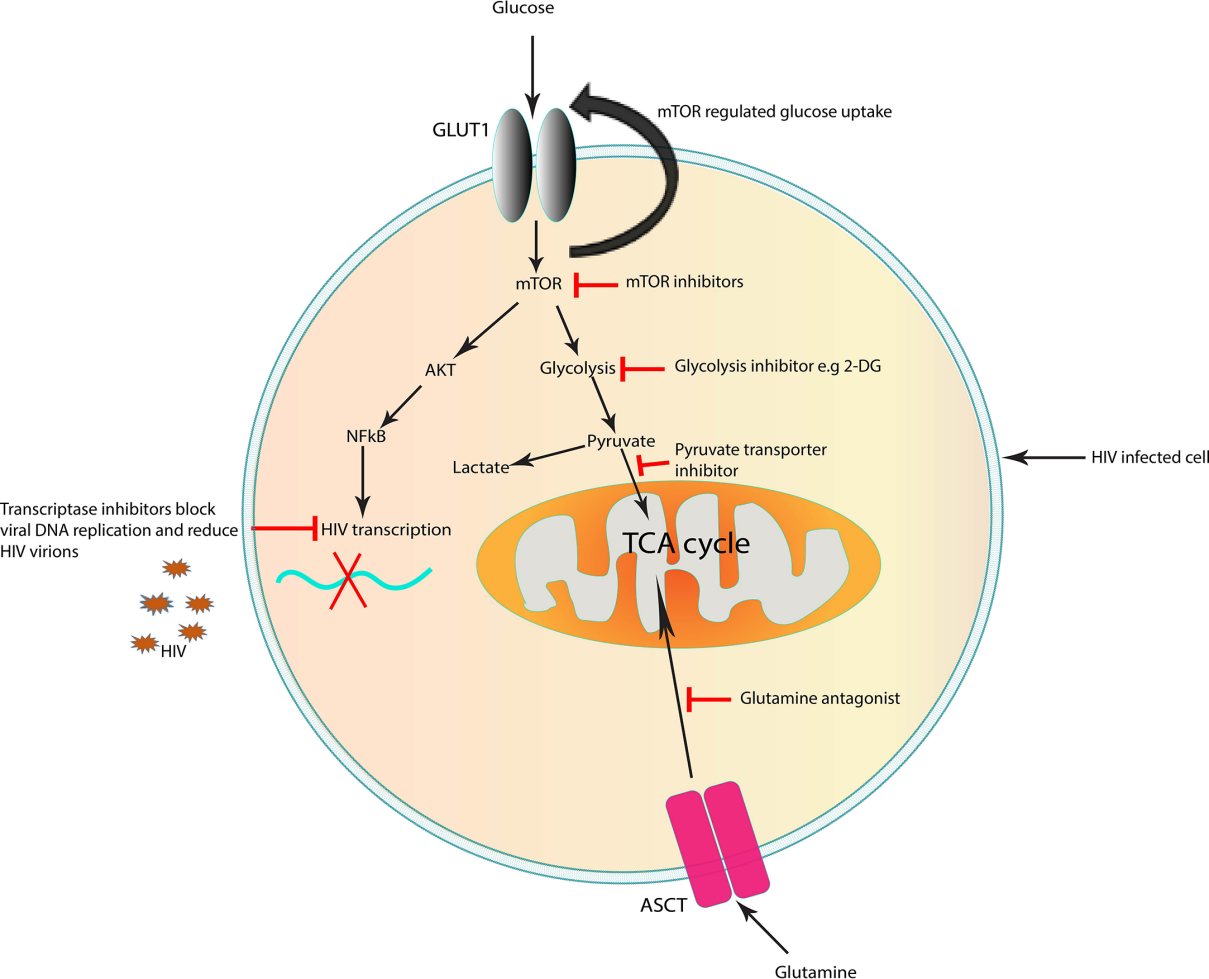
Targets in the cell that could represent a potential strategy to improve immunity for cure in HIV infection. Targeting immunometabolism may affect HIV pathogenesis by inhibiting specific metabolic pathways which are altered by the virus, suppressing inflammation and enhancing immune responses to infection. Targeting glucose and glutamine metabolism can block viral replication. Inhibition of mTOR, which is upregulated in HIV infection, reduce the mTOR-mediated increase in glucose uptake. Suboptimal glycolysis produces less pyruvate which in return controls ROS overproduction and reduces inflammation in HIV infection. The use of therapeutic approaches which improve mitochondrial function may rejuvenate the cells and enhance immune cell mediated antiviral capacity as seen in long-lived memory T lymphocytes. The above strategies may contribute to decreasing the number of infected cells, enhance immune responses to target remaining infected cells and prevention of chronic inflammation. ASCT, amino acid transporter; GLUT1, glucose transporter 1; TCA cycle, tricarboxylic acid cycle; mTOR, mechanistic target of rapamycin. Potential targets which block early steps in HIV replication.

The growing body of evidence of HIV and/or ART-mediated metabolic dysregulation has formed the foundation for the possibility of cellular metabolism as a potential target for complimentary approaches for HIV cure strategies (**Figure 2**) (141, 157). As discussed earlier HIV exposure induces a decrease in the levels of acyl carnitines. Several studies have shown the potential benefits of carnitines supplements as therapy for HIV/ART-induced metabolic dysregulations and mitochondrial dysfunction (158–161). Use of carnitine among PLWHA has been shown to improve symptoms of lactic acidosis, reduce serum triglyceride levels and delay CD4+ T lymphocyte apoptosis in HIV infected adults (159–162). Although these studies were in adult populations, carnitine supplementation may also be beneficial to the paediatric populations.

HIV infection has been associated with increased glycolysis and glutaminolysis in infected lymphocytes (20, 141). Because activated T lymphocytes are largely dependent on glycolysis to fulfil their energy requirements for proliferation and function, these cells are particularly sensitive to a dysregulated metabolism. The plasticity of immune cells such as T and macrophages in their varied energy source during HIV infection has prompted studies on the effects of glutamine/glutamate inhibitors on HIV replication and reservoir size (85, 163).

Several *in vitro* studies have demonstrated the effect of glycolysis and glutaminolysis inhibitors on immune cells functions, HIV infection, replication and reservoir size. Metabolic inhibitors of the enzymes involved in energy metabolism have been demonstrated to reduce HIV infection by inhibiting glycolysis and glutaminolysis (164). Reduced HIV infection is observed in CD4+ T lymphocytes when glycolysis and glutaminolysis are inhibited. Glutamine deprivation resulted in a higher reduction in HIV infected cells compared to glucose deprivation (163). Culturing HIV-infected CD4+ T lymphocytes with poor glycolysis substrates, such as galactose or 2-deoxyglucose, decreases both the cell proliferation and the release of virions in the culture supernatant when compared to culturing in the presence of glucose (72, 165). This indicates a reduced viral latency and replication respectively in a glycolysis-limited environment which is a vulnerability that can be targeted in novel metabolic inhibitors for HIV cure (**Figure 2**). However, given the important role of glycolysis in T lymphocyte functioning, the extent of glycolysis inhibition may decrease T lymphocyte efficacy in eliminating the reservoir and the host defences against other pathogens.

The mammalian (mechanistic) target of rapamycin (mTOR) regulates growth in response to nutrients levels and cellular stress can lead to an increased AMP/ATP ratio, which in turn promotes the phosphorylation and activation of adenosine monophosphate-(AMP)-activated protein kinase (AMPK) (166). Activation of AMPK promotes glucose uptake, glycolysis, fatty acid uptake and fatty acid oxidation and at the same time inhibiting anabolism (gluconeogenesis, synthesis of glycogen, fatty acids and triglycerides) to maintain energy (167). Thus, another potential novel strategy is to optimize immune activity is the treatment with metformin, a drug prescribed to diabetics (168). It is an indirect inhibitor of mTOR which functions by targeting the mitochondrial respiratory chain complex 1 resulting in decreased ATP to ADP ratio (169, 170). This in turn activates AMPK to phosphorylate the raptor subunit on mTOR and alters glycolysis in T lymphocytes (171, 172). The drug has been shown to normalize mitochondrial dysfunction in CD4+ T lymphocytes (168).

The mechanism of action of Metformin in non-diabetic PLWHA is based on this drug’s ability to normalise mitochondrial function through improving ATP production and providing an anti-inflammatory environment, in the process restoring the effective functioning of immune cells (172, 173). Furthermore, the viral reservoir and replication inhibition through Metformin-mediated glycolysis inhibition may play a role in the “block and lock” HIV cure strategy (141). Combining metformin and ART may assist in limiting viral reservoirs. In a recent LILAC pilot study, treatment with metformin among PLWHA showed a preferential activation or phosphorylation of Th17-polarized CCR6+ CD4+ T lymphocytes (171). This subset of CD4+ T lymphocytes is highly targeted by the HIV virus and implicated in the blood and colon viral reservoirs among PLWHA. Interestingly, in the same study treatment with Metformin reduced inflammation and the HIV RNA/HIV DNA ratios indicating a reduced viral transcription (171). It is yet to be explored if there is a synergy between metformin and latency reversal agents, an approach which could be used in combination with ART for viral reservoir eradication.

## Conclusion

Early life exposures to HIV and ART pose a risk on the health outcomes of children born to women living with HIV when compared to those born to healthy women. The impact of these factors on immune cell metabolism and the development of the immune system remain poorly described. There is need for long-term follow-up of these children to monitor clinical implications of metabolic dysregulations. A deeper understanding on how optimal cell metabolism is a central in the functioning of the immune system is critical for exploring alternative novel and safer immunotherapies for HIV cure.

## Author contributions

HM wrote the first draft. All authors were involved in manuscript revisions and approved the final draft.
